# Principles of musculoskeletal sport injuries for epidemiologists: a review

**DOI:** 10.1186/s40621-024-00507-3

**Published:** 2024-05-27

**Authors:** Chinchin Wang, Steven D. Stovitz, Jay S. Kaufman, Russell J. Steele, Ian Shrier

**Affiliations:** 1 Department of Epidemiology, Biostatistics and Occupational Health, McGill University, Montreal, Canada; 2 Centre for Clinical Epidemiology, Lady Davis Institute, Jewish General Hospital, 3755 Côte Ste-Catherine Road, Montreal, QC H3T 1E2 Canada; 3 Department of Family Medicine and Community Health, University of Minnesota, Minneapolis, USA; 4 Department of Mathematics and Statistics, McGill University, Montreal, Canada; 5 Department of Family Medicine, McGill University, Montreal, Canada

**Keywords:** Sport injuries, Musculoskeletal system, Epidemiology, Strains and sprains

## Abstract

**Background:**

Musculoskeletal injuries are a common occurrence in sport. The goal of sport injury epidemiology is to study these injuries at a population level to inform their prevention and treatment.

**Main body:**

This review provides an overview of musculoskeletal sport injuries and the musculoskeletal system from a biological and epidemiologic perspective, including injury mechanism, categorizations and types of sport injuries, healing, and subsequent injuries. It is meant to provide a concise introductory substantive background of musculoskeletal sport injuries for epidemiologists who may not have formal training in the underlying anatomy and pathophysiology.

**Conclusion:**

An understanding of sport injuries is important for researchers in sport injury epidemiology when determining how to best define and assess their research questions and measures.

## Background

Sport and physical activity are crucial to maintaining a healthy lifestyle for people of all ages. Their wide-ranging benefits include prevention of chronic diseases, reduced morbidity and mortality, and improved mental health (Warburton et al. 2006). However, participation in sports and physical activity can also result in injury. Referred to as “sport injuries”, they occur most commonly to the musculoskeletal system that allows the human body to move. Sport injuries can result in morbidity, predispose to further injuries, and decrease subsequent activity due to time lost during recovery or reduced desire to be active (Emery and Pasanen 2019). By studying the epidemiology of sport injuries, we can inform their prevention and management at a population level.

Epidemiologists conducting research in the sport injury field may not have formal training in the anatomy and pathophysiology of sport injuries and the musculoskeletal system. This review aims to provide a concise introductory overview of musculoskeletal sport injuries for epidemiologists, covering (1) definition of a sport injury from biological and epidemiological perspectives; (2) common categorizations of sport injuries and subsequent injuries; (3) a summary of the musculoskeletal systems and the injuries occurring to specific tissues and organs; (4) principles of healing and rehabilitation of sport injuries.

## Main text

### What is a sport injury?

An injury is generally considered a sport injury when it occurs in relation to participation in sport or physical activity. Sport injuries occur most commonly to the musculoskeletal system (Patel and Baker 2006) and as such, we focus on musculoskeletal sport injuries in this review. However, sport participation can also result in injuries to other organ systems such as the neurological system (e.g. concussions, spinal cord injuries and peripheral nerve injuries), cardiovascular system (e.g. arrhythmias), and other systems (Toth et al. 2005; Maron and Pelliccia 2006). In this section, we provide definitions of injury from both the biological and epidemiological perspectives.

#### Biological perspective

**Fig. 1:**
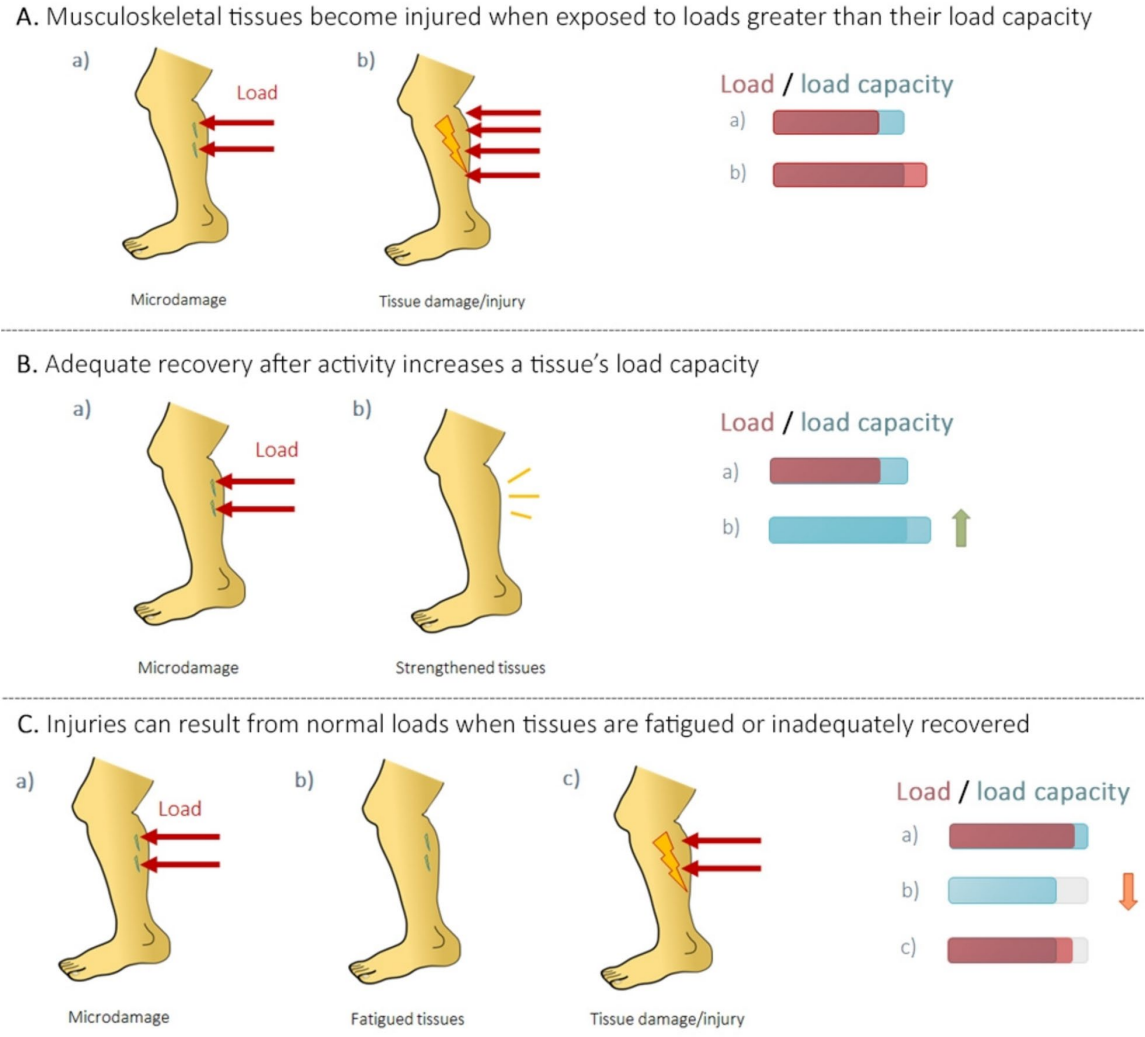
Relationship between a musculoskeletal tissue’s load capacity, load, and injury. The red coloured bars represent load on a tissue, while the blue coloured bars represent the tissue’s load capacity. **A** Tissues exposed to loads lower than their load capacity experience microdamage (panel **a**), while tissues exposed to loads greater than their load capacity experience immediate tissue damage and injury (panel **b**). **B** Given sufficient recovery, microdamage from loads below a tissue’s capacity (panel **a**) will result in strengthening and increased load capacity (panel **b**). **C** Tissues without sufficient recovery (panel **a**) have a decreased load capacity (panel **b**) which can lead to immediate tissue damage and injury even with normal loads (panel **c**)

Biologically, injuries are broadly defined as tissue damage (Bartlett and Bussey 2013). When an individual performs activity, their body is exposed to various forces. For simplicity, we will refer to the sum of these forces as load (Bartlett and Bussey 2013). Load causes tissues to undergo deformation. Upon deformation, tissue cells try to keep or restore themselves to their original state, causing an internal resistance known as stress (Bartlett and Bussey 2013; Gómez-González et al. 2020; Bahr et al. 2012a). Loads beyond a tissue’s load capacity (or load tolerance) will cause excessive stress leading to tissue damage (Fig. 1A) (Bartlett and Bussey 2013; Bahr et al. 2012a).

Tissue damage can occur to varying extents. Large loads beyond a tissue’s load capacity will cause immediate tissue damage. The amount of damage can usually be visualized without a microscope, and may result in physical symptoms (e.g. pain) and limitations of tissue function (Bartlett and Bussey 2013; Bahr et al. 2012a). Healing involves structural changes, where damaged tissue may be repaired or replaced with scar tissue (Baoge et al. 2012). Smaller loads that are close to or minimally above a tissue’s load capacity can cause microdamage or microtrauma, referring to microscopic tears or cracks within a cellular membrane that can be viewed using traditional light microscopy (Bartlett and Bussey 2013; Bahr et al. 2012a; Radasch 1999; Stauber et al. 2020; Lieber 2018). While generally considered an injury in a biological sense, when microdamage is isolated, tissues are usually able to restore tissue integrity without scar tissue, and without most of the physical symptoms of injury (Bahr et al. 2012a). Given sufficient recovery time, structural adaptations in response to microdamage lead to increased tissue strength and load capacity (Fig. 1B) (Wiesinger et al. 2015; Bahr et al. 2012b). However, repeated microdamage without sufficient recovery can result in injury, limiting tissue function and resulting in physical symptoms and scar tissue formation (Fig. 1C) (Bartlett and Bussey 2013; Bahr et al. 2012a; Baoge et al. 2012).

Damage can also be restricted to the internal structure of a tissue cell, which might be only visible using electron microscopy (Lieber 2018). This includes damage at the cell cytoskeleton level (Butterfield 2010; Gefen and Weihs 2016). The cytoskeleton is responsible for cellular structure and stability, and plays crucial roles in cell movement, division, and intracellular transport (Gefen and Weihs 2016). Damage to the cytoskeleton might impair transport of important molecules, leading to decreased cell function (Gefen and Weihs 2016). This damage may or may not be considered an injury. For instance, concussion injuries usually involve a decrease in neuronal brain function without visible microscopic damage (Signoretti et al. 2011). While the underlying mechanisms for the decreased function are not fully understood, this is consistent with the hypothesis of cell cytoskeleton damage (Park et al. 2022).

Finally, fatigue is the loss of tissue strength upon repeated loading and associated microdamage. While not considered an injury in itself, it results in decreased load capacity, which can make a tissue more susceptible to injury (Bartlett and Bussey 2013). Muscle fatigue specifically refers to a reduction in the maximal force or ability to maintain maximal force of the muscles due to repeated use, and is reversible with rest (Enoka and Duchateau 2008). It occurs due to impairments in the contractile proteins and structures that allow muscles to generate force, which can be considered as damage at the cell cytoskeleton level (Westerblad et al. 1993, 1990).

#### Epidemiologic perspective

The International Olympic Committee consensus definition of injury for surveillance and epidemiologic studies is “tissue damage or other derangement of normal physical function due to participation in sports, resulting from rapid or repetitive transfer of kinetic energy” (Bahr et al. 2020). This is distinct from their definition of illness, which is a complaint or disorder where the primary mode does not involve the transfer of kinetic energy (Bahr et al. 2020). Illnesses include disorders resulting from the loss of vital elements (e.g. dehydration) (Bahr et al. 2020), or from the external environment (e.g. heat stroke) during sport, among others.

Injuries often present as pain or other physical symptoms such as aches, soreness, stiffness, or deformities that affect normal physical function (Hainline et al. 2017; Clarsen et al. 2013; Bolling et al. 2019). As such, injuries are sometimes recorded as any patient-reported symptom or complaint of the musculoskeletal system due to physical activity (Bahr et al. 2020; Clarsen et al. 2020). However, the perception of pain can differ between individuals based on age, sex, and level of activity (Hainline et al. 2017; Tesarz et al. 2012; Racine et al. 2012). Further, injuries can occur without pain (e.g., microdamage), and pain can be present independent of tissue damage (Hainline et al. 2017). Therefore, the same underlying biological damage in different individuals may not be similarly defined as an injury.

Some athletes and researchers use a more restricted definition of injury compared to the consensus definition. Many athletes (particularly elite) consider pain and other physical symptoms as a normal part of sport participation (Bolling et al. 2019; Verhagen et al. 2021; Wiese-bjornstal et al. 1998). They perceive an injury as a condition that must preclude them from performing at their normal or optimal level, beyond the experience of pain, which results in altered or missed participation from sport (Bolling et al. 2019; Verhagen et al. 2021).

Common definitions of injury in epidemiologic studies are “any complaint”, encompassing all injuries whether the complaint is symptom- or performance-based; “medical attention injuries”, or injuries where medical attention was sought; and “time-loss injuries”, or injuries causing the athlete to be unable to complete a current or future activity session (Bahr et al. 2020). Medical attention injuries may or may not limit an athlete’s ability to participate in sport, and not all time-loss injuries may be reported, nor require or result in medical attention (Bahr et al. 2020). The optimal definition for a sport injury depends on the research question of interest. For example, researchers may be interested in tissue damage as evidenced by microscopy, a medical diagnosis, a patient-reported complaint, or time lost from sport participation.

Although diagnosing injuries is beyond the scope of this article, a positive diagnostic test for an injury could occur due to (1) an actual injury; (2) a false positive test (i.e. test illustrates abnormal morphology when it is normal); or (3) a misunderstanding of what is “abnormal” (i.e. test accurately illustrates abnormal morphology which is not an actual injury). For example, over 30% of individuals over 50 years who have not had back pain (i.e. uninjured) will have disc herniation on a magnetic resonance image (Deyo and Mirza 2016). Epidemiologists must avoid conflating abnormal tests with clinical diagnoses, especially in small, individual clinic-based studies.

### Categorizations of musculoskeletal sport injuries

Musculoskeletal sport injuries are often categorized by characteristics such as their mode of onset, severity, and anatomical and tissue location in research and surveillance. These categorizations can be used to study the prevention or occurrence of specific groups of injuries. Common categorizations are summarized in Table 1 and discussed further below, and in “The musculoskeletal system” section.

**Table 1:** Common categorizations of sport injuries in sport injury epidemiology

Category	Categorization	Definition
Mode of onset	Acute versus overuse	*Acute*: Sudden onset related to a specific inciting event
		*Overuse*: Gradual onset, may or may not be related to a specific inciting event
	Traumatic versus atraumatic	*Traumatic*: Related to a specific traumatic inciting event
		*Atraumatic*: Not related to a specific traumatic inciting event
Severity	Duration of time-loss	Time from which sport participation is ceased until full return to participation
	Duration of symptoms	Time from onset of symptoms (e.g., pain) until symptoms cease
	Acute versus chronic	*Acute*: symptoms present for a short period of time
		*Chronic*: symptoms present for a long period of time
		The period of time distinguishing acute vs chronic often varies according to tissue type and anatomical location
	Severity of symptoms	Self-reported severity of symptoms, assessed using a scale or scoring system
	Functional consequences	Self-reported or clinician assessed functional consequences of injury, assessed using a scale or scoring system
	Amount of tissue damage	Higher degree injuries represent greater severity, although specific definitions may differ depending on the injury type and location
		*1st degree*: Least severe (e.g. minimal damage or tearing with minimal symptoms)
		*2nd degree*: Moderately severe (e.g. visible damage or partial tearing with symptoms)
		*3rd degree*: Most severe (e.g. complete tearing or rupture of tissue)
Anatomical location	Body part or region of injury	*Upper extremity*: Shoulder, upper arm, elbow, forearm, wrist, hand
		*Lower extremity*: Hip, groin, thigh, knee, lower leg, ankle, foot
		*Trunk*: Chest, abdomen, thoracic spine, lumbar spine
		*Head and neck*: Head, neck
Tissue type	Injuries by tissue type	*Bone*: fracture
		*Muscle*: strain
		*Ligaments*: sprain
		*Tendon*: tendinopathy, tendinosis, tendinitis, partial or complete rupture

Different categorizations may use the same terms with different meanings. We refer readers to the text for discussion of the limitations of these categorizations

#### Mode of onset

A common categorization of sport injuries is by their mode of onset or mechanism of injury. Injuries are often categorized as acute versus overuse, although these definitions are not always consistent. Acute injuries have a sudden onset related to a specific event (Bahr et al. 2020). Biologically, this occurs when an undamaged tissue is subjected to a sudden load beyond its load capacity (McBain et al. 2012). One such injury is a broken bone (i.e. fracture) occurring upon a fall. Overuse injuries, on the other hand, occur gradually due to repetitive loading and associated microdamage to a tissue, without a specific inciting event (Bahr et al. 2020). For example, an athlete might develop considerable pain in the calf muscle (i.e. strain) occurring gradually with repeated physical activity that impedes further activity. The term “overuse” implies that these injuries occur due to excessive activity beyond what the tissues are prepared for from previous activity and loading. Instead, we could consider that these injuries occur from previous “underuse” of the tissues, whereby the individual has not been sufficiently active previously and has not developed the load capacity within their tissues to handle this level of activity (Stovitz and Johnson 2006).

Despite these distinctions, there are grey zones and limitations to this categorization. Some injuries with specific inciting events may have been due to underlying “overuse” (i.e. repeated loading and microdamage without sufficient recovery) rather than a sudden load. An injury may occur if the damaged tissue is subjected to a load that would normally be tolerated if not for the microdamage and reduced load capacity (Fig. 1C) (Bahr et al. 2020). While these injuries occur acutely, their underlying mechanism is consistent with that of overuse.

Another closely-related categorization that is sometimes used in the literature is traumatic (similar to and often used as a synonym for acute) or atraumatic (a.k.a. non-traumatic, similar to overuse) (Merkel and Molony 2012). Like acute injuries, traumatic injuries have a sudden onset related to a specific event. Atraumatic injuries occur without a specific inciting event. Injuries occurring alongside a specific event but that are due to underlying microdamage are considered traumatic under this categorization. For instance, a bone can be weakened due to repetitive activity and associated microdamage without any symptoms. The bone might fracture due to a sudden force, such as pushing off during a sprint race. This might be considered an acute injury because it occurred alongside a specific inciting event, but atraumatic because there was no direct trauma.

#### Injury severity

Injuries can also be described by their severity. Severity may have different meanings for different athletes (e.g., the need for medication or surgery) (Mountjoy et al. 2015), but is often defined as the duration of injury, particularly duration of time lost from participation (Bahr et al. 2020). Time loss is assessed from the date that an athlete begins to have altered or missed participation from sport (which may not be the date of injury onset from a biological standpoint), until the date that they are fully able to participate (Bahr et al. 2020). Time loss can underestimate severity if an athlete returns to sport before the injury is resolved, or overestimate severity if an athlete does not resume their normal participation upon healing (e.g., because their fitness and performance is not at a competitive level) (Bahr et al. 2020).

A similar measure to injury duration is the duration of symptoms. While defined as the amount of time that symptoms are present, it is sometimes dichotomized as acute versus chronic. Under this categorization, acute injuries refer to recent injuries, while chronic injuries refer to injuries where symptoms have been present for an extended period of time. For example, some categorize back pain as chronic when symptoms have been present longer than 12 weeks, and as acute when symptoms have been present for less than 12 weeks (Flint et al. 2014; Chou et al. 2014). Some authors use chronic as a synonym for overuse when describing mode of onset (Lavallee and Balam 2010); however, these are separate concepts (Bahr et al. 2020). Symptom duration may be longer than time loss duration if an athlete returns to full participation with lingering symptoms, or shorter if an athlete does not resume their normal participation at the time their symptoms cease.

Injury severity can also be described by the severity of symptoms, functional consequences, or a composite score (i.e. patient-reported outcome measure), as self-reported by athletes (Westerblad et al. 1993; Clarsen et al. 2013; Chou et al. 2014) or assessed by a clinician. Severity can also be assessed through functional tests. These measures are often assessed using scales or scoring systems (Bahr et al. 2020; Clarsen et al. 2020; Stevenson et al. 2001).

Finally, strain (muscle) and sprain (ligament) injuries are often graded as 1st, 2nd, or 3rd degree based on amount of tissue damage and/or clinical symptoms, with 1st degree being the least severe and 3rd degree being the most severe (Bartlett and Bussey 2013; Järvinen et al. 2000; Noonan and Garrett 1999). However, we note that certain injuries are graded based on factors other than severity of symptoms or tissue damage. These categorizations are reviewed in “The musculoskeletal system” section.

#### Injury anatomical location

Injuries are often grouped by their anatomical location for summary purposes. These groups may be broad (e.g., upper extremity, lower extremity, trunk) or specific (e.g., knee, lower leg, ankle) depending on the context (Habelt et al. 2011). While reporting specific anatomical locations is recommended for injury surveillance programs (Bahr et al. 2020), different injuries to the same location can have very different clinical presentations and outcomes. Although specific locations are important for clinicians who are recommending treatment, epidemiologists often focus on broader categories. These broader categories result in larger sample sizes with potentially greater generalizability, although they have limitations when considering specific injuries (Bahr et al. 2020; Mountjoy et al. 2015).

### The musculoskeletal system and its injuries by tissue type

The musculoskeletal system is made up of distinct tissues that function together to provide shape, stability, and movement to the human body. It plays a central role in the ability for humans to do sport and physical activity (Wind et al. 2005).

The major connective tissues of the musculoskeletal system are bone, cartilage, skeletal muscle, tendons, and ligaments. Bones are the structural basis for the human skeleton (Klenerman 2015). The attachment points of adjacent bones are known as joints. Joints allow for movement of bones with respect to each other (Klenerman 2015). Joints contain cartilage, and are surrounded by ligaments, which are tissue bands that physically attach bones to other bones (Frank 2004). Muscles pass over joints, and are attached to two different bones by tissue bands called tendons (Klenerman 2015). Muscles can be thought of as elastics that shorten (i.e., when contracting) and lengthen (i.e., when relaxing). When a muscle shortens, their attachment sites to each bone come closer together. In general, this leads to movement at the joint (Klenerman 2015).

The following section expands on each location and tissue, and summarizes the injuries that can occur to them.

#### Bones

Bones make up the human skeleton, which provides structure and support for the body (Klenerman 2015). They provide a rigid attachment point for muscles, which allow bones to move (Klenerman 2015). Bones also protect the internal organs, produce blood cells, and store and release minerals and fats (Klenerman 2015; Florencio-Silva et al. 2015).

#### Bone composition

Bones are comprised of two types of bone tissue: cortical, and cancellous. Cortical bone is dense and forms a protective outer layer around cancellous bone, which is spongey and responsible for absorbing the load transmitted to bones (Bartlett and Bussey 2013; Radasch 1999; Klenerman 2015). The thickness of cortical bone and the relative distribution of cortical and cancellous bone tissue differs between different locations within a bone and between bones (Radasch 1999). Bones are surrounded by an outer fibrous sheath called the periosteum. The periosteum contains nerves and blood vessels, and plays a role in bone remodelling both regularly and after injury (Klenerman 2015; Maia Ferreira Alencar et al. 2020).

In the long bone of the limbs, the end of a bone is termed the epiphysis, and primarily consists of cancellous bone (Klenerman 2015). The main shaft of a bone is the diaphysis, and consists primarily of cortical bone (Klenerman 2015). The metaphysis lies between the diaphysis and epiphyses, and consists primarily of cancellous bone (Klenerman 2015). Between the metaphysis and the epiphyses is the epiphyseal plate (growth plate) in children, which is a region of bone growth in long bones such as the tibia (shin bone) or femur (thigh bone) (Jorge et al. 2021). While the epiphyseal plate is composed of cartilage during childhood and adolescence, it calcifies into bone tissue after growth has completed (Radasch 1999; Klenerman 2015). Damage to the epiphyseal plate can result in the slowing or stopping of growth for the affected bone, which can lead to angular deformities or asymmetry in lengths of the lower limbs (Caine et al. 2006; Jones et al. 2017).

#### Bone injuries

Bones respond to load by thickening in mass and strengthening (Klenerman 2015). However, forces beyond the load capacity may result in injury to the bone, called a fracture (Radasch 1999). Among athletes, fractures generally occur after a sudden traumatic event such as a fall, contact with another athlete, or contact with an object (e.g. ball travelling with speed) (Court-Brown et al. 2008).

Stress fractures are distinct from regular fractures in that they do not arise from a single traumatic event, but rather repeated exposure to load (i.e., overuse injury) (Bahr et al. 2012a; Saunier and Chapurlat 2018). Bones are normally in a state of remodelling, where bone tissue breaks down (resorption) and is replaced by new tissue (Saunier and Chapurlat 2018; Hughes et al. 2017). Remodelling increases in response to load and associated microdamage (Saunier and Chapurlat 2018; Hughes et al. 2017). Without adequate recovery time, more bone tissue is resorbed than deposited, creating stress fractures that appear as small cracks (Saunier and Chapurlat 2018; Hughes et al. 2017). While most stress fractures will heal with adequate rest and rehabilitation (Milner 2008), some may require surgery (Shindle et al. 2012). Continued loading onto a stress fracture without adequate recovery may result in a complete fracture (Shindle et al. 2012).

Two special types of fractures that occur in children are greenstick and buckle fractures. Greenstick fractures only affect one side of the bone, creating a crack that does not extend through the entire bone and causing bending of the bone rather than a full break (Noonan and Price 1998). This may be due to increased cartilage content and compliance of young bones compared to adult bones, or because the periosteum sheath surrounding a bone is more elastic in children than in adults, decreasing the likelihood of complete fractures (Maia Ferreira Alencar et al. 2020). Greenstick fractures occur most commonly after a fall (Noonan and Price 1998). Buckle fractures, also referred to as torus fractures, occur due to compression of cortical bone that creates a bulge on one side of the bone, without a full break (Sharp and Edwards 2019). These fractures are common among children and are generally simple to treat through immobilization (Sharp and Edwards 2019).

#### Muscles

Muscles are the core component of the human muscular system, which is responsible for generating force and movement (Frontera and Ochala 2015). While there are three types of muscle (smooth, cardiac, and skeletal), only skeletal muscles are responsible for movement of the human body (Frontera and Ochala 2015).

#### Muscle composition

Skeletal muscle tissue is comprised of individual muscle fibres (Klenerman 2015). The patterns in which the muscle fibres are organized help determine the strength and velocity of the muscular contraction (Bahr et al. 2012a; Klenerman 2015). Muscle is the tissue that is the most capable of strengthening in response to load (Bahr et al. 2012a). This occurs primarily through hypertrophy, in which muscle fibres increase their cross-sectional area (Bahr et al. 2012a). Skeletal muscle tissue also contains structures that convey information about muscle tension and position (Macefield and Knellwolf 2018).

#### Muscle injuries

There are several types of muscle injuries. Muscle tissue will become damaged when the applied load exceeds its load capacity, either through a single event or repetitive microdamage. These injuries are referred to as strains (or pulled muscles by the general public) when caused by stretching or contraction forces (Bahr et al. 2012a). Strains are the most common sport injury (Bahr et al. 2012a; Järvinen et al. 2000). The amount of damage can range from tearing of a few muscle fibres with minimal loss of strength (1st degree) to visible partial tearing of the muscle tissue (2nd degree) to a complete tear/rupture of the muscle (3rd degree) (Järvinen et al. 2000; Noonan and Garrett 1999). Damage to muscle tissue may also damage the structures within the tissue responsible for sensing tension and position through the same mechanisms. For instance, a stretching of the muscle will also stretch these structures. As such, balance and position sense are often disrupted in muscle strains (Kröger 2018).

Muscles can also be injured by compressive forces that exceed the load capacity, typically through a direct blow to the muscle. These injuries are referred to as contusions (Bahr et al. 2012a; Beiner and Jokl 2001). Contusions are often associated with rupture of blood vessels, causing internal bleeding that results in a bruise (Bahr et al. 2012a; Beiner and Jokl 2001). Internal bleeding can lead to various clinical consequences, including some conditions that result in permanent disability (Bahr et al. 2012a; Beiner and Jokl 2001).

One particular consequence of unaccustomed activity is delayed onset muscle soreness (DOMS), which is sometimes used as a proxy for injury (Drew and Finch 2016). DOMS presents as soreness, stiffness, or pain that follows 1–3 days after unaccustomed activity (Bahr et al. 2012a; Cheung et al. 2003). Although frequently studied in animal models, its mechanism is not entirely understood. It is unclear whether findings from studies on DOMS are generalizable to muscle injuries (Lieber 2018).

#### Tendons

Tendons represent connective tissue that physically connects muscles to bones (Klenerman 2015). They enable the transmission of force from muscle to bone, and help stabilize joints (Bahr et al. 2012a; Gracey et al. 2020).

#### Tendon composition

Tendons are composed of dense collagen fibres aligned in the same direction as the muscle fibres (Bahr et al. 2012a; Gracey et al. 2020). Tendons contain few elastic fibres (Bahr et al. 2012a), which causes them to experience only a small change in length for a large amount of force compared to muscle (Gracey et al. 2020). The muscle–tendon interface contains receptors that sense and transmit information about forces within the tendon (Maas et al. 2022).

#### Tendon injuries

Tendons will stiffen when lengthened in response to increasing load but become damaged under excessive load (Gracey et al. 2020). Sudden injuries to the tendons are known as tears or ruptures. These can range in severity from partial tearing/rupture of the tissue to complete tearing/rupture (Bahr et al. 2012a; Gracey et al. 2020).

Tendons can also experience overuse injuries (Bahr et al. 2012a). Repetitive loading and microdamage to tendons result in chronic pain, known as tendinopathy (Bahr et al. 2012a). Tendinopathies include tendinosis in cases with tissue degeneration, and tendinitis in cases with tendon inflammation (Bartlett and Bussey 2013; Bahr et al. 2012a; Bass 2012).

Most musculoskeletal injuries occur at the junction where the muscle joins the tendon (Gimigliano et al. 2021). Although the tendon may be involved, these are generally considered muscle injuries. In general, tendon injuries that are not close to the muscle–tendon junction often have poor blood supply, resulting in longer recovery times for tendon-specific injuries compared to muscle injuries (Sharma and Maffulli 2006; Ahmed et al. 1998).

#### Joints

A joint is the point where two or more bones connect. Joints may provide stability or support movement depending on their type and composition (Klenerman 2015).

#### Joint composition

There are three types of joints that differ in composition and function: fibrous, cartilaginous, and synovial joints.

Fibrous joints are fixed, generally immobile joints comprised of dense collagen rather than cartilage, and are found in the skull among other locations (Miroshnichenko et al. 2018). Cartilaginous joints are joined by fibrocartilage or hyaline cartilage (see “Cartilage” section) and are slightly mobile or immobile (Juneja et al. 2022). The epiphyseal plate in long bones, which connects the diaphysis and epiphyses in childhood and adolescence, is considered a cartilaginous joint (Juneja et al. 2022). They are found where the right pelvis joins the left pelvis, among other locations (Becker et al. 2010).

Synovial joints are the most common joints in the human body and include the major joints of the limbs (e.g. knee, elbow, shoulder) (Klenerman 2015). They are mobile and are comprised of a joint cavity, consisting of the ends of the bones that are covered by articular cartilage (Pacifici et al. 2005; Hui et al. 2012). The joint cavity is surrounded by fibrous tissue known as a joint capsule, and lined with a synovial membrane that secretes fluid to keep the joint lubricated (Klenerman 2015; Hui et al. 2012; Ralphs and Benjamin 1994). The joint capsule seals the joint cavity, keeping synovial fluid inside, and provides stability by limiting joint movements and preventing bones from separating (Ralphs and Benjamin 1994). As synovial joints allow for movement, they are the most commonly implicated in sport injuries (Bartlett and Bussey 2013).

Joints can be categorized by the types of movement they allow. Joints can move in three planes: sagittal (longitudinal), frontal (coronal), and transverse (axial) (Milner 2008). Movement in the sagittal plane is seen from the side of the body (e.g. knee flexion and extension), movement in the frontal plane is seen from the front of the body (e.g. hip abduction and adduction), and movement in the transverse plane is seen from above (e.g. hip rotation) (Milner 2008). Hinge (uniaxial) joints are a type of synovial joint where most movement occurs in a single plane, and are found in the elbow and knee (Fornalski et al. 2003; Hefzy and Grood 1988). Biaxial joints often experience movement in two planes, and include the metacarpophalangeal (finger knuckle) joints (Batmanabane and Malathi 1985). Multiaxial joints often experience movement in three planes, and include the hip and shoulder joints (Bowman et al. 2010; Peat 1986).

#### Joint injuries

Joint dislocations are a common injury that occur when the bones that connect at the joint are displaced, resulting in immediate pain and limited range of motion (Skelley et al. 2014; Hodge and Safran 2002). Dislocations typically occur through a sudden traumatic force such as a collision or fall, and occur commonly in the shoulder and elbow among athletes (Skelley et al. 2014; Hodge and Safran 2002). Dislocations can cause damage to the ligaments, cartilage, and bones (Skelley et al. 2014). Dislocations are treated by physical manipulation of the joint back into its normal location, followed by a recovery period often involving immobilization to heal tissue damage (Skelley et al. 2014; Hodge and Safran 2002). Recurrent dislocations in some joints (e.g. shoulder) are common among athletes (Skelley et al. 2014).

Subluxations are partial joint dislocations where the connecting bones do not completely separate. Unlike full dislocations, subluxations sometimes spontaneously “relocate” to their original position without physical manipulation (Aronen 1986). Joint injuries also include specific injuries to cartilage and ligaments. These injuries are covered in their respective sections.

#### Cartilage

Cartilage is an important connective tissue mainly present in joints (Klenerman 2015). Cartilage is weaker and more flexible than bone. However, it is still weight-bearing and resilient (Klenerman 2015). There are three types of cartilage: hyaline, fibrocartilage, and elastic (Klenerman 2015). Elastic cartilage, which is present in the ear and larynx, is not considered a component of the musculoskeletal system (Pollard et al. 2017).

#### Hyaline cartilage composition

Hyaline cartilage is the most common cartilage in the human body (Krishnan and Grodzinsky 2018). It does not contain any nerves or blood vessels, and is limited in its ability to repair itself following damage (Sophia Fox et al. 2009). It is found inside joints covering the ends of adjacent bones, where it is referred to as articular cartilage (Krishnan and Grodzinsky 2018).

Articular cartilage is a highly specialized tissue that reduces friction and provides a smooth surface for movement at the joints (Sophia Fox et al. 2009). It redistributes pressure across bones to minimize high pressure point loads that could cause bone swelling and injury (Sophia Fox et al. 2009). Its nutrition comes from molecules dissolved in the normal joint fluid (synovial fluid). As the joint moves, synovial fluid circulates and distributes nutrients (Sophia Fox et al. 2009). When joint movement is restricted, for instance due to injury or casting, cartilage nutrition is impaired (Sophia Fox et al. 2009; Wang et al. 2013). Immobilizing a joint is one method of creating osteoarthritis in animals (Palmoski and Brandt 1981).

#### Hyaline cartilage injury

Similar to bone, hyaline cartilage can be injured through a single traumatic event (Buckwalter 2003). Furthermore, extensive damage to articular cartilage with insufficient repair leads to unequal redistribution of forces within the joint. One possible consequence of articular damage is post-traumatic osteoarthritis, a condition characterized by joint pain, dysfunction, and malalignment (Buckwalter 2003). Although articular cartilage itself is not visible on X-rays, insufficient repair may lead to a decreased cartilage thickness, causing the two bones of a joint to appear much closer together than normal on an X-ray. This is called joint space narrowing, and is an important sign for clinically meaningful osteoarthritis (Chan et al. 2008).

#### Fibrocartilage composition

Fibrocartilage is a stronger and denser type of cartilage than hyaline cartilage (Klenerman 2015). Unlike hyaline cartilage, it contains nerves and blood vessels, but only at its periphery (Bahr et al. 2012a). It is typically found in larger joints, and functions to absorb and distribute forces more evenly across bones (Bahr et al. 2012a). Fibrocartilage tissue is present in the meniscus of the knee, and the labrum in the hip and shoulder (Bahr et al. 2012a; Klenerman 2015). It also forms part of the intervertebral discs that lie between the bones of the lumbar spine (Klenerman 2015).

#### Fibrocartilage injury

Damage to fibrocartilage occurs in meniscal and labral injuries (tears). Acute meniscal tears occur due to trauma to the knee, and can occur in isolation, or alongside injury to ligaments (Lohmander et al. 2007). Degenerative meniscal tears are more common with increasing age and increased loading (e.g. weight-bearing activities) (Adams et al. 2021; Howell et al. 2014). As we age, the meniscus becomes weaker and more susceptible to tears with low loads. Labral tears in the hip and shoulder can also occur from trauma or degeneration from repetitive loading (Keener and Brophy 2009; O’Reilly et al. 2022; Banerjee and Mclean 2011). Hip labral tears are associated with certain types of abnormal hip morphologies (Banerjee and Mclean 2011).

#### Ligaments

Ligaments represent connective tissue that physically connects bones, spanning a joint (Frank 2004). They are often just local prominent thickenings of the joint capsule tissue that run from one bone to the other, with a different tissue composition (Ralphs and Benjamin 1994). However, some ligaments exist inside or outside of the joint capsule (Ralphs and Benjamin 1994). Their primary function is to stabilize joints and prevent excessive movement at the joint (Frank 2004; Milner 2008). However, ligaments also play an important role in proprioception as they contain nerve endings that convey information about joint position and movement that are necessary to coordinate contractions by different muscles during movement (Bahr et al. 2012a). Ligaments stretch out in response to low amounts of load, but will resist movement when pulled tight in response to further load, thus preventing further movement of the joint (Frank 2004; Milner 2008). However, ligaments will tear if stretched too far, causing injury (Frank 2004; Gracey et al. 2020).

#### Ligament composition

Ligaments are primarily composed of dense collagen fibres, with only small amounts of elastic fibres (Frank 2004; Milner 2008). They are generally strong and stiff, with limited elasticity (Milner 2008). The degree of stiffness differs by the relative composition of collagen versus elastic fibres and other components (Solomonow 2004). The stiffness of a ligament also increases as the ligament is stretched (Frank 2004).

#### Ligament injuries

While ligaments have a relatively high load tolerance, excessive load will cause damage and injury to the ligaments and other joint structures (Frank 2004; Gracey et al. 2020). Injuries to ligaments are referred to as sprains (Bartlett and Bussey 2013). These can range in severity from some tissue damage with minimal symptoms (1st degree), partial tear of the ligament (2nd degree), to complete tearing/rupture and separation of the damaged ends of the ligament (3rd degree). Grade definitions and terminology can differ by injury type. For instance, there are three ligaments that are implicated in a lateral ankle sprain. Some categorizations use 3rd degree ankle sprain to refer to the complete tearing of each ligament, while some consider a 3rd degree ankle sprain to mean all three ligaments are damaged (Wolfe et al. 2001; Lacerda et al. 2023). Surgery may be recommended for some complete ligament tears but not others (Beynnon et al. 2005; Miyamoto et al. 2009; Stone et al. 2021). Because damage to a ligament may damage local nerves, proprioception, balance and position sense are often disrupted in sprains (Bahr et al. 2012a).

Nearly all ligament injuries occur due to a single event (Bartlett and Bussey 2013). However, ligaments and other joint structures may also experience microdamage when subjected to repetitive loading (Frank 2004; Solomonow 2004). This microdamage is usually asymptomatic, but may affect joint stability and predispose individuals to other injuries (Bahr et al. 2012a).

### Healing and rehabilitation of injury

#### Biological and clinical perspective

Injury healing occurs in three overlapping phases: (1) inflammation; (2) repair or regeneration; and (3) remodelling (Pieters et al. 2021). Briefly, inflammation causes damaged cells and tissues to degenerate (Pieters et al. 2021; Bigham-Sadegh and Oryan 2015). Tissue repair or regeneration replaces damaged cells with new cells. Finally, the repaired or regenerated tissue is remodelled to regain optimal strength and function in a process that can take months to years (Pieters et al. 2021; Bigham-Sadegh and Oryan 2015). The specific healing process of an injury depends on the type of tissue that is damaged and the degree of damage. Bone heals by a regenerative process, whereby the healed tissue is the same as the original bone tissue (Bahr et al. 2012a). Other musculoskeletal tissues heal by a regenerative process when there is less severe tissue damage or microdamage. However, severe tissue damage heals by a repair process, where the healed tissue is a scar tissue rather than the original tissue (Bahr et al. 2012a; Liu et al. 2018; Leong et al. 2020). While scar tissue is initially weak, its strength increases during the repair and remodelling process until it is close to that of the original uninjured tissue (Järvinen et al. 2000). Extensive scar tissue formation due to tissue bleeding and inflammation may result in decreased tissue strength and increased risk of recurrent injuries (discussed below) (Bahr et al. 2012a).

Tissue strength can be increased during the repair and remodelling process by applying progressive loads that cause microdamage and subsequent adaptation, but remain below the load capacity for injury (Bahr et al. 2012b; Magnusson and Kjaer 2019; Kiviranta et al. 1988; Tipton et al. 1975; Liphardt et al. 2024; Sun 2010; Killian et al. 2012). Overloading may lead to further tissue damage and disrupt the healing process. For instance, low loads during a recovery period may still be large enough to cause further tissue damage because of the reduced load capacity (Bahr et al. 2012b). Just as overloading may cause tissue damage, underloading or extensive immobilization may prevent tissues from strengthening and cause tissue atrophy (Magnusson and Kjaer 2019; Liphardt et al. 2024; Sun 2010; Fournier 2015).

Clinicians usually prescribe rehabilitation therapy for injuries. Rehabilitation refers to restoring the tissue to its preinjury state, and involves many components that are not always well described in studies (Wade et al. 2010). Most injury rehabilitation programs start with reducing pain and preventing excess bleeding and inflammation, reducing scar tissue formation. Additionally, injuries often result in decreased range of motion, strength and proprioception. Therefore, exercises including those for balance, strengthening, and stretching are often prescribed to specifically address these limitations (Fournier 2015). Other components of rehabilitation may include electronic modalities (e.g. ultrasound), manual therapy (e.g. massage, mobilizations, manipulations), and prevention education. Finally, patients are recommended to gradually return to participation in sport once they are largely symptom-free and have regained adequate strength to minimize injury recurrence (Fournier 2015).

#### Epidemiological perspective

Injury healing can be assessed under different definitions, which will affect calculated injury durations. Ideally, an injury is considered healed when the athlete is able to return to their previous amount of activity without pain. When detailed data are available, healing date can be determined from clinical records or self-report of symptoms. Alternatively, researchers might use the date of last treatment for injury, under the assumption that treatment is only provided while an injury remains unhealed (Hamilton et al. 2011). However, the decision to stop treatment has subjectivity (Hamilton et al. 2011). As such, clinical data may not always minimize error or bias in the measurement of healing.

Unfortunately, detailed data are often unavailable and researchers often operationalize the healing date of an injury as the date of full return to participation or play in sport (Bahr et al. 2020; Hamilton et al. 2011). However, many athletes return to activity while they are still symptomatic, which could result in measurement error if utilizing return to play or even medical clearance date as the healing date. Epidemiologists should recognize that return to participation decisions can vary between athletes, coaches, and clinicians, and may not necessarily reflect biological healing (Bahr et al. 2020; Hamilton et al. 2011).

#### Subsequent and recurrent injuries

Initial injuries may predispose to subsequent injuries due to muscle imbalances, deficits in strength and proprioception, or changes in biomechanics (Fulton et al. 2014). Subsequent injuries to the same location account for a considerable proportion (10–25%) of all injuries (Hamilton et al. 2011).

Researchers must consider how to define and account for subsequent injuries, particularly in the longitudinal follow-up of athletes. Subsequent injuries generally refer to injuries that occur after an initial index injury. Subsequent injuries to a different body part are considered “subsequent new injuries” (Hamilton et al. 2011). Subsequent injuries to the same body part but a different tissue type are called “local injuries”. Finally, subsequent injuries to the same body part and tissue type are called “recurrent injuries”.

Recurrent injuries can be exacerbations or re-injuries. An “exacerbation” is a worsening of an index injury that was not fully healed or recovered (Bahr et al. 2020; Hamilton et al. 2011). A “re-injury” is a recurrent injury that occurs to the same location and tissue as an index injury that was fully healed or recovered (Bahr et al. 2020). Recurrences are sometimes further categorized by the time that they occurred following healing of the index injury (early: within 2 months; late: within 2–12 months; or delayed: more than 12 months) (Hamilton et al. 2011).

These categorizations have limitations. For instance, it can be unclear whether a subsequent injury is related or not related to the initial injury (Shrier and Steele 2014). Further, the definition of healing within an epidemiologic study will affect whether an injury is considered a re-injury or an exacerbation. This will in turn affect the overall injury count, risk, rate, and length of time loss. Consider an individual who suffers an index injury on January 1st and returns to participation on January 10th but continues to experience symptoms and receive medical treatment. They then experience a worsening of symptoms on January 30th. If healing is defined as return to participation, the individual will be considered as having had two injuries (an index injury and a subsequent/recurrent injury), each with a separate length of time loss with the sum being the total time lost. If healing is defined as date of last treatment or by cessation of symptoms, the individual will be considered as having one injury (an index injury and an exacerbation), with a longer time loss (equal to the total time lost). While there is no consensus for the optimal definition of healing, researchers would benefit from clearly defining their outcomes. Further, when synthesizing and interpreting findings from multiple studies, researchers should ensure that aggregated results use similar definitions.

## Conclusion

Sport injuries are a concern for anyone participating in physical activity. Applying epidemiologic methods can greatly contribute to determining how to best prevent and treat sport injuries and their related morbidities. Understanding what constitutes a sport injury from a biological and epidemiologic perspective is important for researchers in these fields, who must determine how to best define and assess their research questions and measures.

## Data Availability

Not applicable.
